# Differences in Valsa Canker Resistance and Its Physiological Mechanisms Among Various Apple Rootstock–Scion Combinations

**DOI:** 10.3390/plants15172689

**Published:** 2026-09-01

**Authors:** Jiaxu Zhou, Chenghui Li, Hongjian Li, Yige Xu, Xiumei Zhang, Ying Liu, Lihong Han, Nianwen Yu

**Affiliations:** Liaoning Institute of Pomology, Yingkou 115009, China; zhoujiaxu568@163.com (J.Z.); yigoxu@163.com (Y.X.);

**Keywords:** rootstock combinations, apple, Valsa canker, anatomical structure, photosynthetic performance

## Abstract

To compare resistance to apple Valsa canker across scion–rootstock combinations and to identify key evaluation indicators and superior resistant combinations, we established 15 scion–rootstock pairings using ‘Yueyanghong’, ‘Harlikar’, and ‘Qiufuhong’ as scions and Qingzhen No.1, Qingzhen No.2, *M. baccata* Borkh., *M. robusta* Rehd., and *M. hupehensis* Rehd. as rootstocks. We assessed resistance under natural field infection conditions. We measured plant growth indices, leaf photosynthetic parameters, anatomy of one-year-old shoots, and mineral nutrient contents for each combination. We then performed correlation analysis with the disease index to identify relationships between these indicators and disease occurrence under diseased conditions and to clarify their physiological basis. Finally, we comprehensively evaluated grafting compatibility and disease resistance among scion–rootstock combinations to identify combinations that might reduce Valsa canker severity and the associated predictive indicators. Results indicated that combinations using ‘Yueyanghong’ as the scion had the lowest overall disease index. In particular, ‘Yueyanghong’/Qingzhen No.2 showed the best graft compatibility (FCC = 2.10, RSR = 1.10) and the most favorable comprehensive disease-resistance profile. ‘Yueyanghong’/*M. hupehensis* Rehds exhibited the highest photosynthetic rate. By contrast, ‘Harlikar’/Qingzhen No.2 and ‘Qiufuhong’/*M. baccata* Borkh displayed relatively high disease indices and were classified as highly susceptible under the experimental field conditions. Lower disease index correlated with higher leaf net photosynthetic rate and with higher intercellular CO_2_ concentration. Combinations with low relative electrical conductivity retained greater membrane-system stability. A lower disease index was positively associated with higher potassium (K) content, while higher iron (Fe), manganese (Mn), and zinc (Zn) contents correlated with increased disease index. Finally, lower disease index corresponded to greater shoot phloem thickness and higher cell density. In conclusion, using the disease index as a quantitative measure and integrating multidimensional indicators, plant growth, photosynthetic physiology, mineral nutrition, and shoot anatomical structure, allows preliminary evaluation of compatibility and disease resistance across scion–rootstock combinations.

## 1. Introduction

Apple (*Malus domestica* Borkh.) is the largest fruit crop in China, with the greatest cultivation area and output worldwide; it plays an essential role in agricultural economic growth, rural revitalization, and increases in farmers’ incomes. Apple Valsa canker, caused by Valsa Mali, is a destructive branch disease that spreads rapidly, persists for long periods, and is difficult to control, and it affects all major apple-producing regions in China. The pathogen mainly infects the trunk and main-branch xylem, causing bark rot and reduced tree vigor; in severe cases, whole-plant death can occur. Over the years, it has caused sustained yield losses and substantial economic damage, and it remains a persistent challenge for disease prevention and control in apple production [1]. This disease not only reduces apple yield and fruit quality but also shortens orchard renewal cycles and raises production costs, posing a serious threat to the sustainable development of the apple industry. Current control measures for Valsa canker rely on chemical pesticides, manual scraping of lesions, and pruning of diseased or weak branches. However, Valsa Mali hyphae can establish latent infections deep within tree tissues, and chemical agents seldom penetrate fully into the niches where the pathogen resides [2]. Manual removal of diseased tissue cannot completely eliminate pathogen-infected cells, so lesion recurrence is likely. Therefore, developing efficient, environmentally friendly strategies to control Valsa canker and elucidating apple resistance mechanisms to Valsa Mali have substantial practical value for sustaining the apple industry.

As a central technical component of apple cultivation, rootstock–scion combinations modulate scion growth, fruiting characteristics, and environmental adaptability [3,4]. Variation in disease resistance among rootstock–scion combinations has therefore become a primary industry concern. Rootstocks regulate stomatal aperture, transpiration, and water-use efficiency, which in turn affect growth, fruit quality [5,6], sugar accumulation [7,8], yield, and flowering time [9,10]. They also provide anchorage, facilitate soil nutrient uptake, and enhance resistance to biotic and abiotic stresses [11,12]. Previous studies have shown that in ‘Fuji’ apples, rootstock type influences the structure and composition of the rhizomal microbial community. Dwarf rootstocks exhibit greater bacterial richness and diversity in the roots and harbor a larger proportion of beneficial microorganisms, while Valsa Mali is detected at lower levels than in arbor rootstocks [13]. Zhou et al. reported that phloridzin accumulation is inversely correlated with resistance to Valsa canker. The pathogen can use phloridzin as a carbon source to boost toxin production. The phloridzin biosynthetic pathway modulates apple resistance to Valsa Mali by regulating cell-wall lignin and pectic polysaccharide deposition and by altering the balance between salicylic acid and reactive oxygen species. Plants with reduced phloridzin biosynthesis display compact tissues and thicker bark [14]. Liu et al. showed that treating apple branches with the biocontrol bacterium Bacillus velezensis D4 up-regulated genes in three defense signaling pathways: salicylic acid, ethylene, and jasmonic acid. The bacterium antagonized pathogens by inhibiting cell-wall biosynthesis and inducing accumulation of reactive oxygen species [15]. The cited studies indicate that rootstock–scion combinations can alter Valsa Mali infection and colonization by modulating phenylpropanoid metabolic flux and the accumulation of secondary metabolites in trees. Activation of defense signaling pathways may also synergize with these rootstock–scion-mediated changes to regulate disease resistance.

Identifying disease-resistant apple germplasm and screening tolerant rootstock–scion combinations are critical research priorities. Yet few studies have systematically examined field incidence of Valsa canker or the regulatory mechanisms that differ among rootstock–scion combinations. Identifying disease-resistant apple germplasm and screening tolerant rootstock–scion combinations are critical research priorities. Yet few studies have systematically examined field incidence of Valsa canker or the regulatory mechanisms that differ among rootstock–scion combinations. Previous studies have shown that net photosynthetic rate influences tree vigor and can indirectly affect disease resistance by modulating the biosynthesis of defense-related compounds and oxidative stress responses [16]. Therefore, beyond performing field resistance assessments for Valsa canker, this study systematically measured photosynthetic parameters, chlorophyll content, and mineral nutrient concentrations across different rootstock–scion combinations. The objective was to elucidate the relationships between these physiological indicators and Valsa canker resistance, thereby providing a physiological basis for selecting superior, disease-resistant rootstock–scion combinations.

Most studies have focused on identifying Valsa Mali pathogens, elucidating infection and pathogenic mechanisms, and developing disease-control strategies. Systematic field-based evaluations of rootstock–scion combinations for resistance to Valsa canker and their correlations with plant physiological metabolism and branch anatomical structure remain limited. A systematic understanding remains lacking regarding how rootstock–scion combinations influence tree resistance to Valsa canker by modulating leaf photosynthesis, mineral nutrient accumulation, and branch anatomical structure. Likewise, few studies have screened suitable rootstocks for specific scion cultivars to satisfy practical field-cultivation needs. Accordingly, dwarfing rootstocks Qingzhen No.1, Qingzhen No.2, and standard rootstocks *M. baccata* Borkh, *M. robusta* Rehd, and *M. hupehensis* Rehd were selected as rootstock materials, while ‘Yueyanghong’, ‘Qiufuhong’, and ‘Harlikar’ were used as scions to establish a total of 15 rootstock–scion combinations for this experiment. Among them, ‘Yueyanghong’ [17] and ‘Qiufuhong’ [18] are new apple cultivars independently bred by the Liaoning Institute of Pomology. We evaluated field resistance to Valsa canker in 15 rootstock–scion combinations. For each combination, we measured photosynthetic parameters, chlorophyll content, mineral nutrient concentrations, and branch anatomical traits. The study aimed to elucidate the physiological mechanisms by which rootstock–scion pairings affect Valsa canker resistance, to identify reliable indicators and assessment methods for canker resistance, and to provide a theoretical basis for selecting superior disease-resistant combinations and for disease-resistant apple cultivation.

## 2. Results

### 2.1. Effects of Different Rootstock–Scion Combinations on Apple Canker Resistance

**Figure 1:**
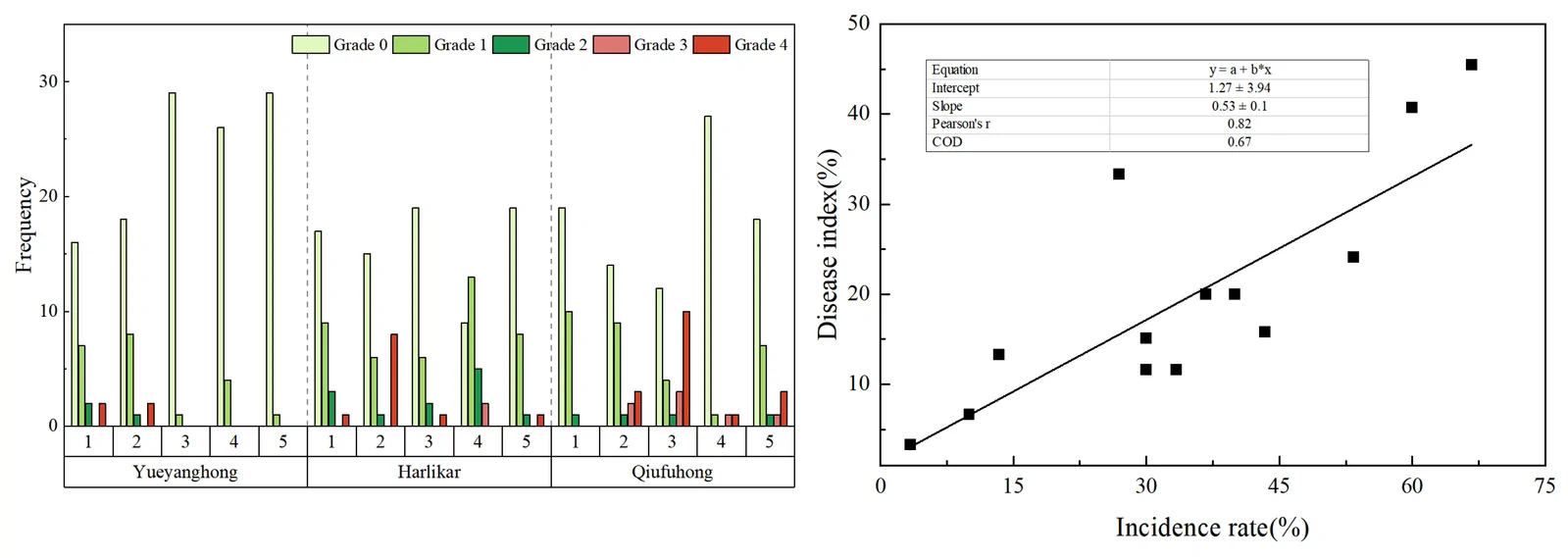
Frequency distribution and correlation analysis of Valsa canker among different rootstock–scion combinations. Note: 1: Qingzhen No.1, 2: Qingzhen No.2, 3: *M. baccata* Borkh., 4: *M. robusta* Rehd., 5: *M. hupehensis* Rehd. * in the figure denotes a multiplication sign.

We performed a correlation analysis between rot disease incidence and disease index across 15 rootstock–scion combinations and found a significant positive relationship (Figure 1). The figure below displays the frequency distribution of rot disease severity. Combinations using ‘Yueyanghong’ as the scion showed strong resistance; except for the Qingzhen No.1 and Qingzhen No.2 combinations, severity in all other ‘Yueyanghong’ combinations clustered in grades 0–1. When ‘Harlikar’ served as the scion, the Qingzhen No.2 combination exhibited higher severity than the others, reaching grade 4, while the remaining ‘Harlikar’ combinations showed similar disease levels. With ‘Qiufuhong’ as the scion, the Qingzhen No.1 and *M. robusta* Rehd combinations performed well in the field, whereas the *M. baccata* Borkh combination had a high frequency of grade 4 disease. Overall, ‘Yueyanghong’ displayed the mildest disease severity, and all its combinations outperformed those of the other scions, indicating strong potential for disease resistance.

### 2.2. Effects of Rootstock–Scion Combinations on Tree Growth

As shown in Table 1, rootstock, scion, and their interaction each had significant effects on plant growth. Rootstock had a comparatively large influence on rootstock diameter, scion diameter, the rootstock–scion ratio, and the field compatibility constant (FCC). Scion significantly affected graft–union diameter, while rootstock did not. The rootstock–scion interaction had an extremely significant effect on rootstock diameter and a significant effect on graft–union diameter, but it had only minor effects on the rootstock–scion ratio and FCC. Overall, for plant-growth indicators, rootstock effects exceeded those of scion and the rootstock–scion interaction. When ‘Yueyanghong’ served as scion, the Qingzhen No.2 combination performed best. Its rootstock diameter (RD) and FCC value were significantly higher than those of other combinations (*p* < 0.05). The rootstock–scion ratio (RSR) and FCC value were close to 1 and 2, respectively, indicating optimal graft compatibility in the field. Although the *M. baccata* Borkh. combination exhibited the largest scion diameter (SD), its rootstock–scion ratio was significantly lower than those of other combinations (*p* < 0.05), and its FCC value was well below 2, consistent with an excessively large scion and poor field compatibility. When ‘Harlikar’ was used as scion, the rootstock–scion ratios of the *M. robusta* Rehd. and *M. hupehensis* Rehd. combinations were each close to 1. The FCC value for *M. robusta* Rehd. was approximately 2, indicating good graft compatibility. The rootstock–scion ratio and FCC value for Qingzhen No.2 were significantly higher than those for other combinations (*p* < 0.05), although overall tree growth remained weak. When ‘Qiufuhong’ served as the scion, combinations with Qingzhen No.1 and *M. baccata* Borkh. produced larger scion diameters. The Qingzhen No.2 combination exhibited a rootstock–scion ratio near 1 and the highest FCC value, indicating optimal graft compatibility. Overall, FCC values for ‘Qiufuhong’ combinations were lower than those for ‘Yueyanghong’ and ‘Harlikar’ combinations, suggesting relatively poor field compatibility.

### 2.3. Effects of Rootstock–Scion Combinations on Leaf Growth and Physiological Characteristics

#### 2.3.1. Effects on Leaf Growth

As shown in Table 2, Rootstock, scion, and their interaction had only minor effects on leaf growth. Scion significantly affected leaf width, and both scion and the rootstock–scion interaction influenced leaf area. As shown in the following table, leaf thickness (LT) did not differ significantly among rootstock–scion combinations, indicating that rootstock type had little effect on LT. When ‘Yueyanghong’ was the scion, the Qingzhen No.1 combination showed clear advantages; leaf length (LL, 9.58 cm), leaf width (LW, 5.35 cm), and leaf area (LA, 36.81 cm^2^) exceeded those of other combinations (*p* < 0.05). Its leaf area was 15.97% larger than that of the *M. robusta* Rehd. combination, which was the second best. There were no significant differences in leaf growth among combinations with ‘Harlikar’ as the scion (*p* < 0.05). With ‘Qiufuhong’ as the scion, the *M. hupehensis* Rehd. combination produced greater leaf length and leaf area than other combinations, while the Qingzhen No.2 combination had the greatest leaf width; aside from LL, however, differences among these combinations were not significant.

#### 2.3.2. Leaf Photosynthetic Capacity and Membrane System Stability

Scion significantly influenced leaf intercellular CO_2_ concentration (Ci), relative electrical conductivity, SPAD value, and the photosynthetic performance index, with effects larger than those of the rootstock–scion interaction. As shown in Table 3, Rootstock had only minor effects on leaf photosynthetic parameters. Different rootstock–scion combinations showed significant variation in photosynthetic traits, membrane stability, and chlorophyll content. When ‘Yueyanghong’ was used as scion, combinations with *M. hupehensis* Rehd. and Qingzhen No.2 performed best, exhibiting net photosynthetic rates (Pn) of 16.50 and 15.26, respectively. Relative electrolyte leakage in these combinations was low (0.30–0.32), indicating stable membrane systems, and SPAD values exceeded 60. The combination with *M. robusta* Rehd. showed the lowest Pn and comparatively weak overall performance. When ‘Harlikar’ was the scion, Pn and stomatal conductance (Gs) for combinations with *M. baccata* Borkh. and *M. robusta* Rehd. were significantly higher than those of other combinations (*p* < 0.05). In contrast, the combination with Qingzhen No.2 exhibited the lowest Pn, transpiration rate (Tr), and Gs, along with increased relative electrolyte leakage indicative of greater membrane damage. When ‘Qiufuhong’ served as scion, overall Pn and membrane stability were weaker than in the ‘Yueyanghong’ series. Only the combination with Qingzhen No.1 showed notably high Pn and Tr. The combination with *M. hupehensis* Rehd. had the highest SPAD value, which was significantly greater than those of the Qingzhen No.1 and Qingzhen No.2 combinations (*p* < 0.05).

Table 4 presents a systematic analysis of scion–rootstock interactions on photosynthetic function. The maximum photochemical efficiency of PSII (Fᵥ/F_m_) across all combinations ranged from 0.78 to 0.84, which lies within the normal physiological range and indicates a stable primary light energy conversion efficiency of PSII that was not markedly affected by rootstock. Scions of ‘Harlikar’ showed the highest values of maximum fluorescence (F_m_), variable fluorescence (Fᵥ), and performance index (PI_ABS), reaching a maximum PI_ABS of 6.35, which indicates the strongest PSII electron transport efficiency and overall photosynthetic capacity. Scions of ‘Qiufuhong’ exhibited intermediate PI_ABS values (4.26–4.92) and a clear differentiation in initial fluorescence (F_0_) among rootstocks; the Qingzhen No.1 combination had significantly higher F_0_ than the others (*p* < 0.05). Scions of ‘Yueyanghong’ showed pronounced variation in photosynthetic parameters; the Qingzhen No.1 combination attained the highest PI_ABS, significantly exceeding the *M. baccata* Borkh. combination (*p* < 0.05), while Fᵥ/F_m_ and F_0_ remained stable with no significant differences among combinations (*p* < 0.05).

#### 2.3.3. Leaf Mineral Nutrient Contents

Table 5 presents macronutrient differences among the rootstock–scion combinations. Scion identity had a stronger influence on K and Ca than did rootstock, whereas Mg was primarily controlled by rootstock. Neither rootstock, scion, nor their interaction significantly affected N or P. Across the three scions, ‘Yueyanghong’, ‘Harlikar’, and ‘Qiufuhong’, N and Mg accumulation showed little sensitivity to rootstock, with no significant differences among combinations (*p* < 0.05). P content exhibited rootstock–specific effects only in the ‘Yueyanghong’ scion; the *M. hupehensis* Rehd. combination had a significantly lower P content than the other combinations (*p* < 0.05), while combinations with ‘Harlikar’ and ‘Qiufuhong’ showed no significant differences. The accumulation of certain macronutrients (Ca, K) exhibited clear scion–rootstock interaction specificity. For ‘Yueyanghong’ scions, Ca levels in the Qingzhen No.1 and Qingzhen No.2 combinations were significantly higher than in the *M. hupehensis* Rehd. and *M. robusta* Rehd. combinations (*p* < 0.05). For ‘Harlikar’ scions, Ca levels in the Qingzhen No.1, *M. baccata* Borkh. and *M. hupehensis* Rehd. combinations, and K levels in the *M. baccata* Borkh. combination were significantly higher (*p* < 0.05). For ‘Qiufuhong’ scions, K levels in the Qingzhen No.2 combination and Ca levels in the *M. robusta* Rehd. combination were significantly lower than in the other combinations (*p* < 0.05). Micronutrient accumulation was more strongly governed by rootstock identity, and the patterns differed among scions.

Table 6 summarizes micronutrient differences among rootstock–scion combinations. For ‘Yueyanghong’ scions, the Qingzhen No.2 combination had significantly higher Fe, Cu, and B levels (*p* < 0.05), whereas Mn was higher with *M. hupehensis* Rehd. and *M. robusta* Rehd. rootstocks. For ‘Harlikar’ scions, Qingzhen No.1 and *M. hupehensis* Rehd. combinations showed significantly elevated Fe and Mn (*p* < 0.05), and Qingzhen No.1 also had superior Cu. For ‘Qiufuhong’ scions, the *M. hupehensis* Rehd. combination yielded significantly higher Fe, Mn, and Cu (*p* < 0.05). Across all three scions, B followed a consistent ranking: *M. baccata* Borkh. combination > *M. robusta* Rehd. combination > *M. hupehensis* Rehd. combination.

### 2.4. Effects of Rootstock–Scion Combinations on Branch Anatomical Structure

**Figure 2:**
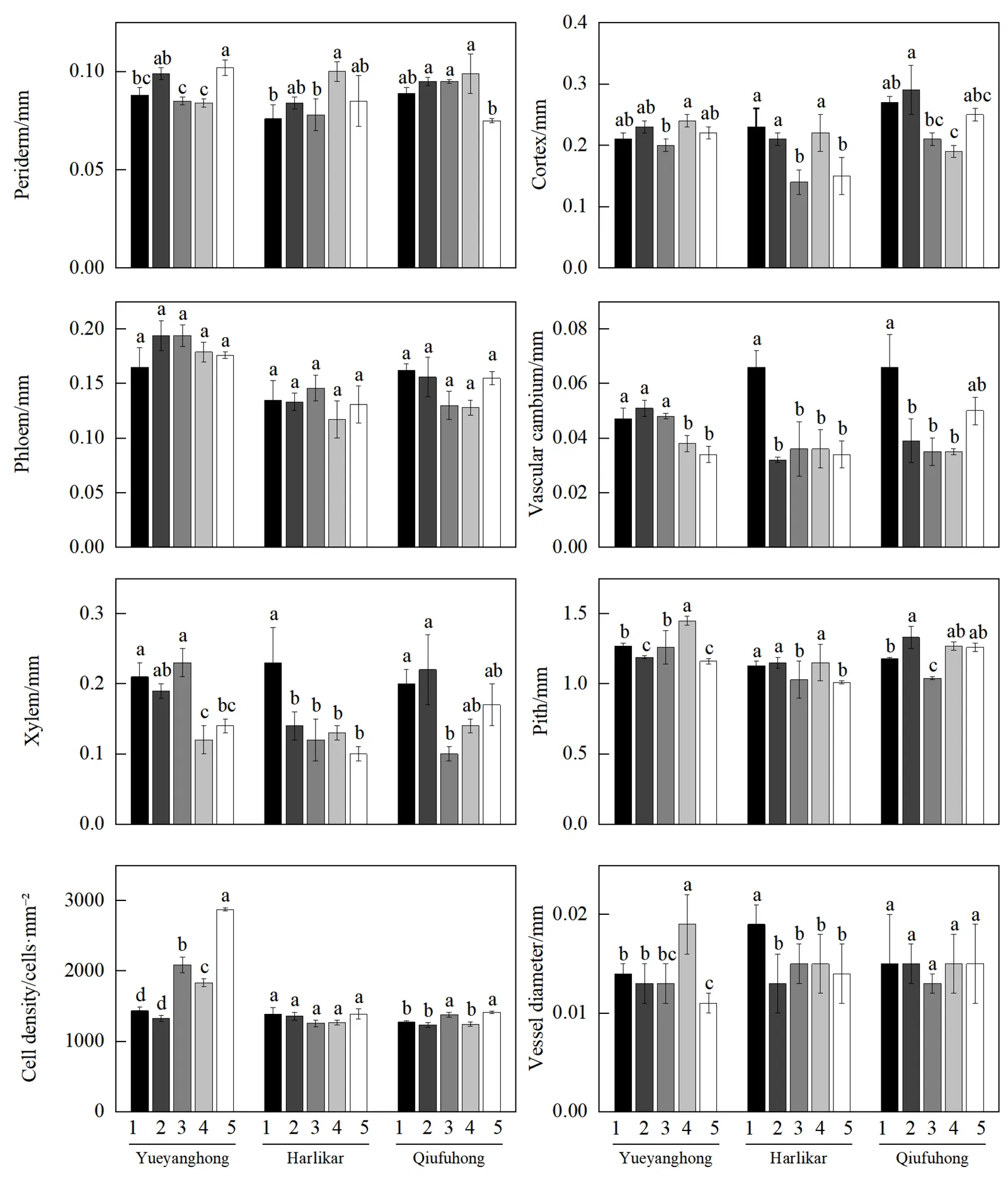
Anatomical structure differences in one-year-old shoots among different rootstock–scion combinations. Note: Lowercase letters indicate significant differences at the *p* < 0.05 level. One-way ANOVA was used for each Scion identifier. 1: Qingzhen No.1, 2: Qingzhen No.2, 3: *M. baccata* Borkh., 4: *M. ro-busta* Rehd., 5: *M. hupehensis* Rehd.

Figure 2 compares anatomical parameters of annual branches across rootstock–scion combinations. The results show that rootstock substantially modified scion anatomy, with variation both among indices within a given scion and among scions for a given index. Phloem thickness varied little among combinations, and ‘Yueyanghong’ scions consistently had greater phloem thickness than other scions. For cortex thickness, the *M. robusta* Rehd. rootstock under ‘Yueyanghong’ scion was 20% higher than the *M. baccata* Borkh. rootstock, while it did not differ significantly from the remaining combinations. Under ‘Harlikar’ scion, combinations with *M. baccata* Borkh. and *M. hupehensis* Rehd. showed lower cortex thickness and differed significantly from the other combinations. Under the ‘Qiufuhong’ scion, the Qingzhen No.2 combination had the greatest cortex thickness, 18.3% higher than the lowest value observed with *M. robusta* Rehd. Periderm thickness followed the pattern Qingzhen No.2 > Qingzhen No.1 across all scions, although the two did not differ significantly. Under the ‘Yueyanghong’ scion, combinations with *M. hupehensis* Rehd. and Qingzhen No.2 produced significantly greater periderm thickness than the other combinations. Under ‘Harlikar’ and ‘Qiufuhong’ scions, the *M. robusta* Rehd. combination yielded the largest periderm thickness and differed significantly from Qingzhen No.1, *M. baccata* Borkh., and *M. hupehensis* Rehd. combinations.

For pith thickness, the *M. robusta* Rehd. rootstock produced the greatest values with the ‘Yueyanghong’ scion, and it also performed best with the ‘Harlikar’ scion, where it was significantly superior to the *M. baccata* Borkh. and *M. hupehensis* Rehd. combinations. With the ‘Qiufuhong’ scion, Qingzhen No.2, *M. robusta* Rehd., and *M. hupehensis* Rehd. combinations exceeded the *M. baccata* Borkh. combination. For cell density, the *M. hupehensis* Rehd. combination under the ‘Yueyanghong’ scion was significantly higher than the others, showing a 116.85% increase relative to the lowest Qingzhen No.2 combination; overall, vigorous rootstocks had higher cell density than dwarfing rootstocks. No significant differences among combinations were observed under the ‘Harlikar’ scion. Under the ‘Qiufuhong’ scion, *M. baccata* Borkh. and *M. hupehensis* Rehd. combinations had significantly higher cell density than the other combinations. Across scions, ‘Yueyanghong’ exhibited the highest pith cell density, suggesting greater canker resistance than ‘Harlikar’ and ‘Qiufuhong’. For vessel diameter, *M. robusta* Rehd. and Qingzhen No.1 combinations were significantly larger under the ‘Yueyanghong’ and ‘Harlikar’ scions, respectively; no significant differences among combinations were found with the ‘Qiufuhong’ scion. The Qingzhen No.1 combination also showed superior xylem and cambium thickness, generally exceeding that of vigorous rootstocks. Among vigorous rootstocks, only the *M. baccata* Borkh. combination with the ‘Yueyanghong’ scion was significantly superior to *M. robusta* Rehd. and *M. hupehensis* Rehd., while other comparisons showed no significant differences.

In conclusion, rootstock type exerts scion-specific and trait-specific control over apple scion anatomy. Qingzhen-series rootstocks generally increase the thickness of lignified tissues, including xylem and cambium, whereas *M. hupehensis* Rehd. and *M. baccata* Borkh. produce stronger responses in traits such as cell density and cortex thickness.

### 2.5. Correlation Analysis Between Disease Resistance and Physiological and Anatomical Indicators

**Figure 3:**
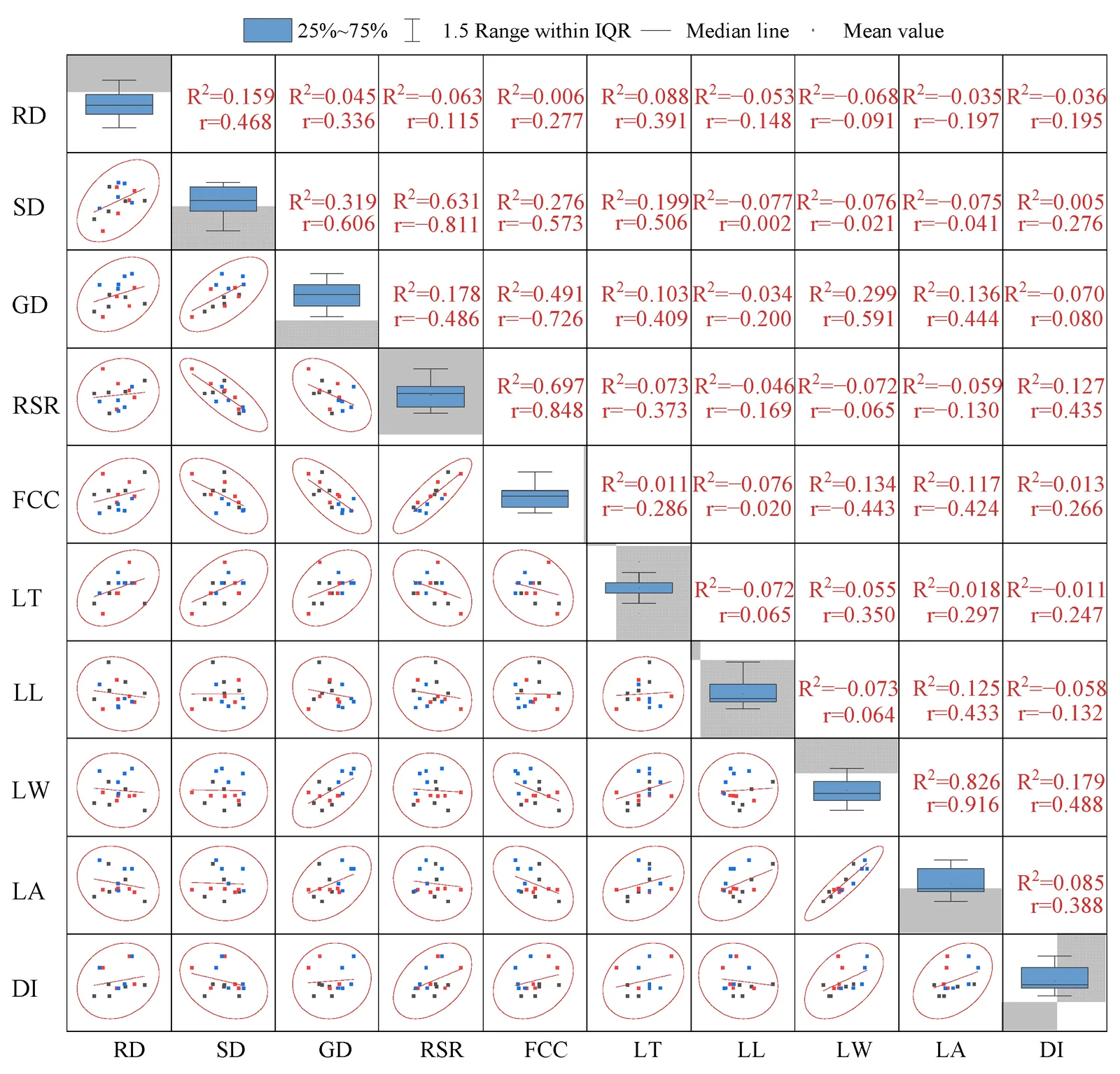
Correlation between disease index and plant growth indices. Note: Circles represent the 95% confidence ellipse, lines represent linear fitting, black dots represent ‘Yueyanghong’, red dots represent ‘Harlikar’, and blue dots represent ‘Qiufuhong’.

Figure 3 shows the correlation analysis between disease index and plant growth indicators. Leaf width correlated positively with disease index, indicating that larger leaf width corresponded to higher disease index. The rootstock–scion ratio (RSR) also correlated positively with disease index, implying that higher RSR associated with greater disease severity. Among synergistic relationships among morphological indicators, scion diameter correlated positively with both rootstock diameter and graft union diameter. Graft union diameter also correlated positively with RSR and the field compatibility constant (FCC). These findings indicate strong growth coordination between aboveground organs and rootstocks and suggest that such traits can indirectly influence disease resistance by modifying RSR. Additional correlations, leaf width with leaf area and leaf length, FCC with graft union diameter, and RSR with FCC, further underscore the intrinsic links among plant morphology, rootstock–scion compatibility, and leaf traits.

**Figure 4:**
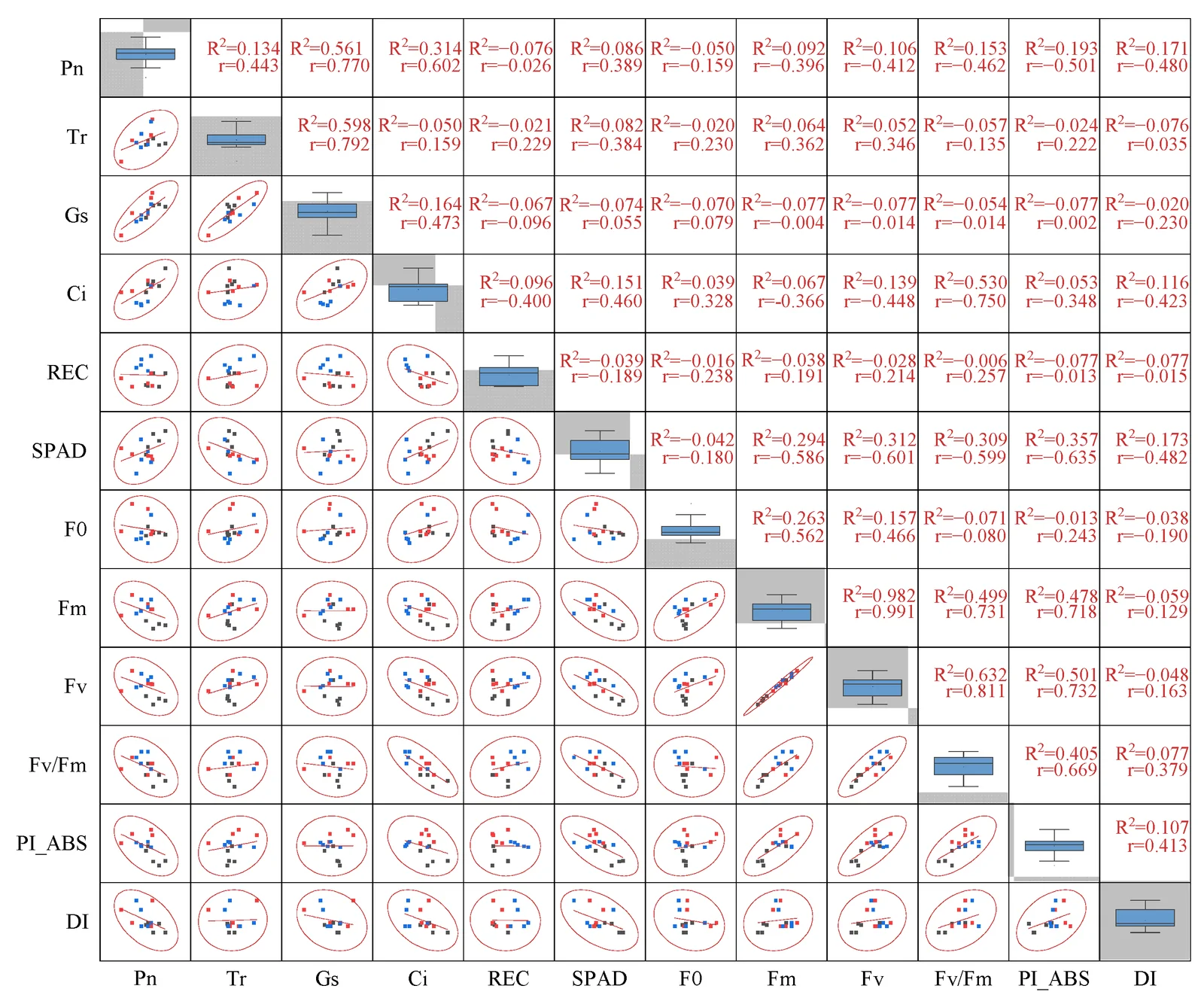
Correlation between disease index and leaf photosynthetic capacity indices. Note: Circles represent the 95% confidence ellipse, lines represent linear fitting, black dots represent ‘Yueyanghong’, red dots represent ‘Harlikar’, and blue dots represent ‘Qiufuhong’.

Leaf gas-exchange parameters displayed clear signatures of synergistic regulation. As shown in Figure 4, net photosynthetic rate (Pn) correlated positively with transpiration rate (Tr), stomatal conductance (Gs), and intercellular CO_2_ concentration (Ci); among these, Gs was tightly associated with Tr. Ci correlated positively with SPAD value, and it correlated negatively with variable fluorescence (F_V_) and the maximum photochemical efficiency of PSII (F_V_/F_m_), which could suppress PSII photochemical conversion efficiency. Chlorophyll fluorescence parameters exhibited very strong internal coherence: initial fluorescence (F_0_) correlated positively with both maximum fluorescence (F_m_) and F_V_, while F_m_ correlated extremely closely with F_V_. Moreover, F_m_ and F_V_ correlated positively with F_V_/F_m_ and the performance index on an absorption basis (PI_ABS). Together, these three metrics reflected PSII functional status and varied in a highly consistent manner. Correlations between photosynthetic parameters and the disease index revealed the response pattern to disease stress: Pn and Ci correlated negatively with the disease index, whereas PI_ABS correlated positively with the disease index, suggesting that PSII may sustain basic photosynthetic function through compensatory regulation under disease stress.

**Figure 5:**
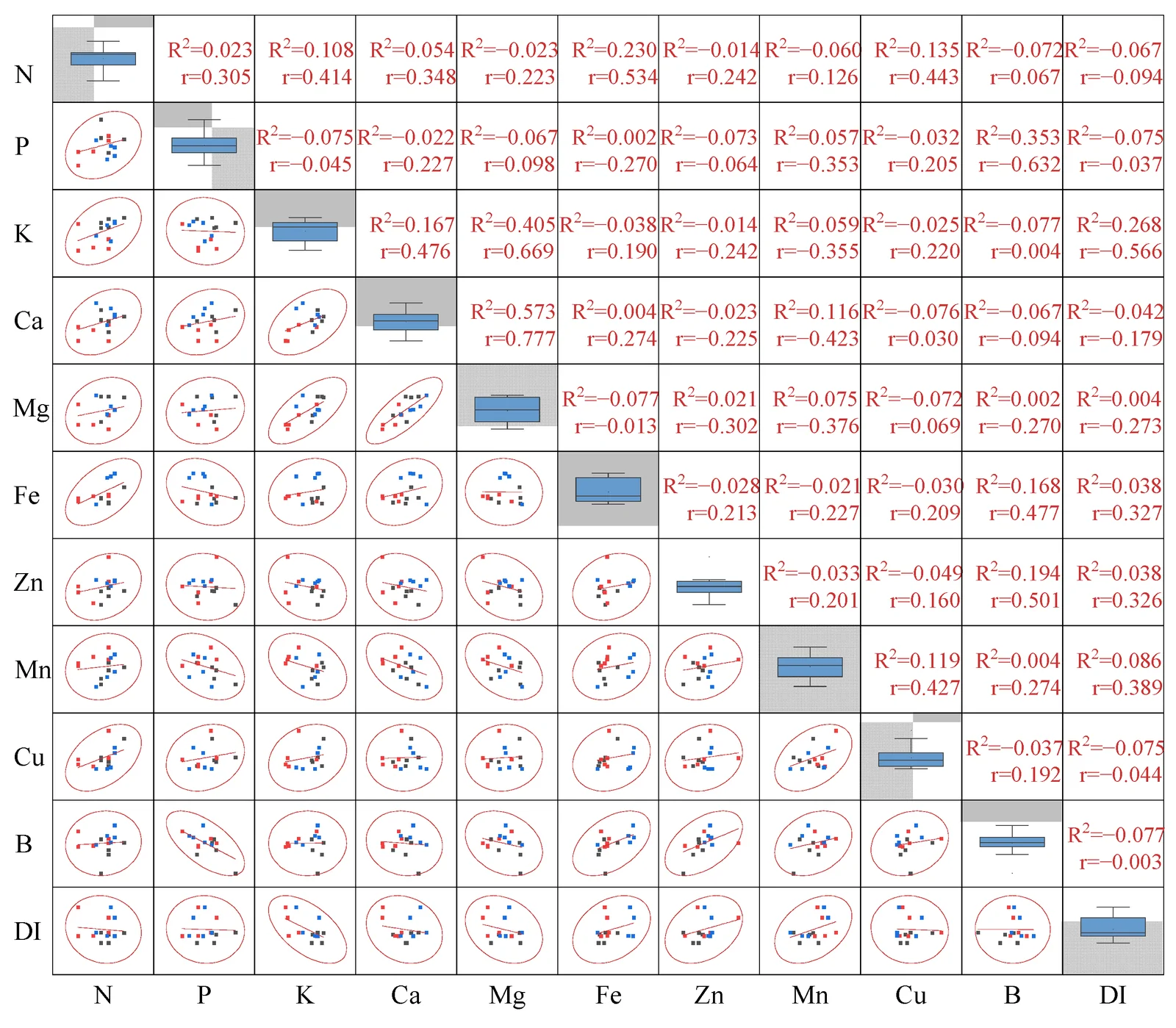
Correlation between disease index and leaf mineral elements. Note: Circles represent the 95% confidence ellipse, lines represent linear fitting, black dots represent ‘Yueyanghong’, red dots represent ‘Harlikar’, and blue dots represent ‘Qiufuhong’.

Significant synergistic relationships were observed among leaf mineral elements. As shown in Figure 5, nitrogen (N) correlated positively with potassium (K), iron (Fe), and copper (Cu). Potassium (K) correlated positively with calcium (Ca) and magnesium (Mg) and correlated negatively with the disease index. Calcium (Ca) correlated positively with magnesium (Mg). Directly associated with the disease index, leaf Fe, manganese (Mn), and zinc (Zn) contents were positively correlated with disease severity, indicating that the disease index increased as these element levels rose. These findings suggest that excessive accumulation of these minerals in leaves may be linked to greater disease severity and that they could serve as potential physiological indicators for early disease warning.

**Figure 6:**
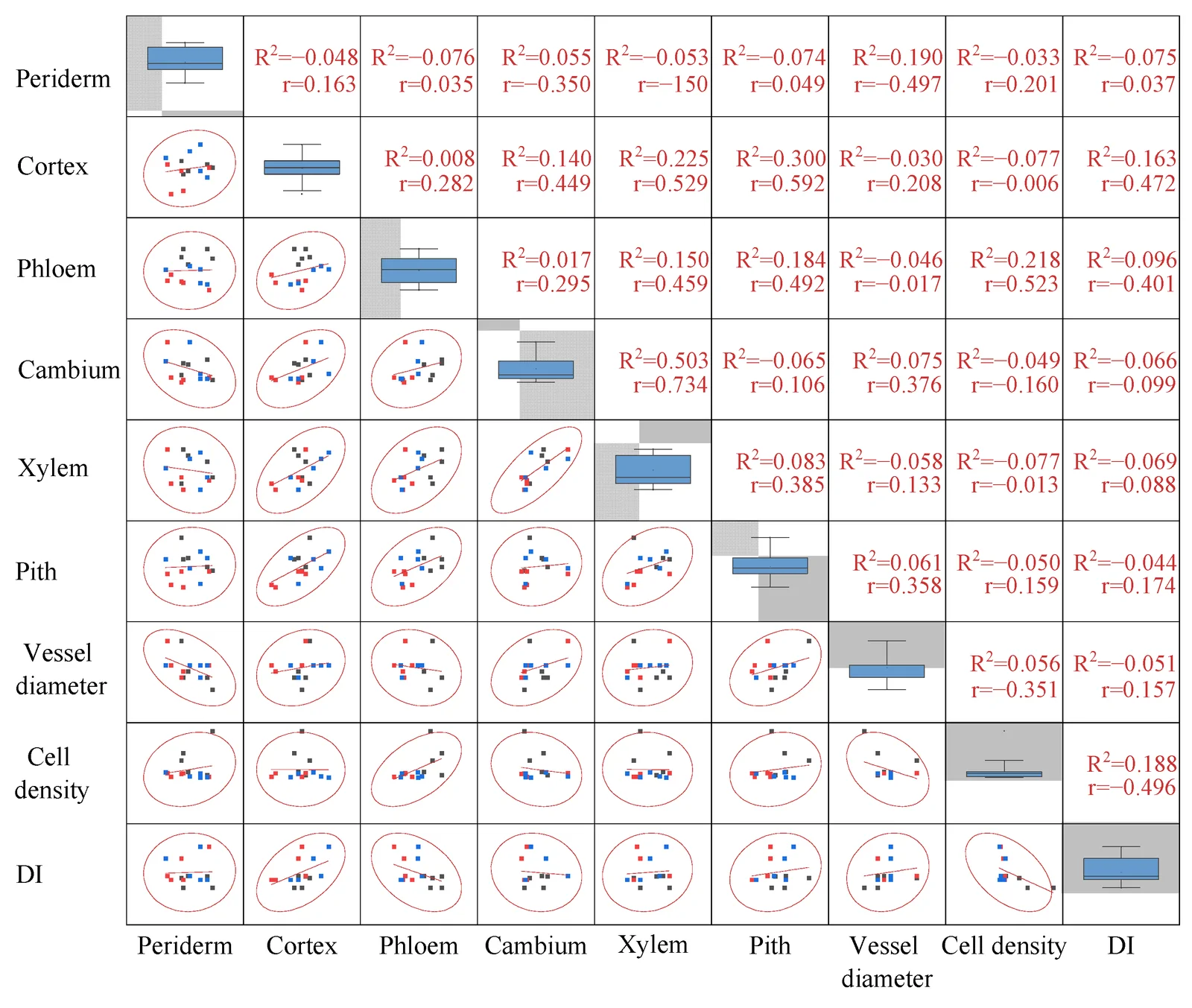
Correlation between disease index and tissue structure. Note: Circles represent the 95% confidence ellipse, lines represent linear fitting, black dots represent ‘Yueyanghong’, red dots represent ‘Harlikar’, and blue dots represent ‘Qiufuhong’.

Figure 6’s correlation analysis shows that periderm thickness was negatively correlated with vessel diameter, indicating that vessel diameter decreased as periderm thickness increased. Cortex thickness was positively correlated with vascular cambium thickness, xylem thickness, pith thickness, and the disease index, with the strongest association observed for xylem thickness. Phloem thickness correlated positively with xylem thickness, pith thickness, and cell density, and correlated negatively with the disease index; thus, increased phloem thickness was associated with greater xylem and pith thickness and higher cell density, while coinciding with a lower disease index. Vascular cambium thickness was positively correlated with xylem thickness, suggesting that thicker cambium promotes xylem expansion. Finally, cell density was strongly negatively correlated with the disease index across all three cultivars tested, implying that cell density may be an important physiological indicator of disease severity.

## 3. Discussion

Rootstocks are central to fruit tree cultivation. They regulate tree growth and fruit quality and modulate scion disease resistance by optimizing root-system absorption, adjusting in-plant metabolism, and reinforcing tissue architecture. Consequently, rootstocks occupy a key position in integrated disease-control strategies for fruit trees [19,20,21]. Apple Valsa canker is among the most destructive stem and branch diseases affecting apple production. It substantially shortens the orchard renewal cycle, increases planting costs, and constrains the high-quality, sustainable development of the apple industry. Consequently, screening superior disease-resistant rootstocks and elucidating the regulatory mechanisms that govern disease resistance in rootstock–scion combinations have become essential technical strategies for preventing and controlling apple Valsa canker. Numerous studies have shown that rootstock markedly influences fruit-tree resistance to Valsa canker. Field monitoring of apple rootstocks by Kairova et al. [22] demonstrated that rootstocks bearing specific resistance alleles effectively resisted pathogen infection, and that rootstock genetic background correlated strongly with disease-resistance phenotypes. Sun et al. [23] showed in grape rootstock work that optimal rootstock–scion combinations enhance stress tolerance by raising antioxidant enzyme activity and preserving cell-membrane stability, providing an important reference for studies of disease-resistance mechanisms in fruit-tree rootstock–scion interactions. We evaluated disease resistance across rootstock–scion combinations assembled from five rootstocks (including *M. robusta* Rehd., *M. baccata* Borkh., etc.) and three apple scion cultivars. The disease index of the ‘Yueyanghong’/*M. hupehensis* Rehd. combination was significantly lower than that of all other tested combinations. This resistance correlated with a thicker xylem and greater pith cell density. In addition, leaf mineral element accumulation in this combination showed distinct favorable profiles. These results align with Kairova et al.’s conclusion [22] that tissue architecture and genetic background together govern plant disease resistance.

Field surveys of disease resistance were conducted at the peak occurrence of Valsa canker in March, while physiological and anatomical measurements used current-year branches and leaves sampled in July. Hou et al. [24] noted that field natural-infection surveys for assessing apple germplasm resistance to ring rot are constrained by environmental conditions and that resistance expression varies with cultivation practices and climatic factors. Infection by Valsa Mali markedly increases plant enzyme activities, peaking 1–2 days after inoculation [25]. Thus, the July measurements likely reflect physiological responses to spring Valsa canker infection rather than inherent cultivar resistance. This experiment aimed to document the growth of newly developing branches and leaves in different rootstock–scion combinations under natural field recovery following spring infection by Valsa Mali. By integrating the disease-resistance survey from March with measurements taken at the full-fruit stage in July, the results offer practical guidance for orchard management.

This study demonstrates that resistance to apple Valsa canker is not governed by a single trait but emerges from the combined effects of rootstock–scion interactions, tissue structure, and physiological metabolism. Because each rootstock–scion combination was planted in spatially contiguous blocks, observed effects cannot be fully separated from local variation in soil properties, drainage, microclimate, or pathogen pressure. Therefore, the main effects of rootstock, scion, and their interaction should be interpreted in the context of this specific field design. In the comparative field evaluation of phenotypic traits among different rootstock–scion combinations, it was found that among these factors, rootstock–scion compatibility provides a fundamental basis for disease resistance. Good compatibility permits normal transport of nutrients and signaling molecules within the tree, preserves physiological homeostasis and vigor, and establishes the conditions necessary to activate plant defense responses. Imbalanced compatibility can substantially reduce a tree’s resistance to stem-branch diseases. Even when certain rootstock–scion combinations perform well on some physiological measures, severe disease symptoms can still develop if compatibility is poor and physiological homeostasis is disrupted. This observation underscores the decisive role of growth-development homeostasis in fruit-tree disease resistance. From an anatomical perspective, the pith cell density of one-year-old branches may represent a resistance-related trait for assessing Valsa canker resistance in rootstock–scion combinations. This study adds anatomical, physiological, and biochemical evidence for Valsa canker resistance in apple rootstock–scion combinations.

Analysis of leaf phenotypic traits across apple rootstock–scion combinations showed that rootstock–scion pairing had limited influence on leaf morphology. Leaf length and leaf thickness did not differ significantly among combinations, and only minor differences occurred in leaf width and leaf area. These findings indicate that leaf morphological traits are primarily determined by the cultivars’ intrinsic genetics and are only weakly affected by rootstock–scion interactions. Compared with the relatively stable traits of leaf morphology, leaf photosynthetic physiology and mineral nutrient accumulation are more responsive to rootstock–scion combinations. These two sets of traits also correlate closely with tree resistance to Valsa canker and can serve as useful auxiliary criteria for assessing the disease-resistance of rootstock–scion combinations. Previous studies have shown that leaf photosynthetic capacity and mineral nutrient metabolism are central regulators of plant disease-defense systems. Wang et al. [26] reported that mineral nutrients enhance disease resistance via multiple mechanisms: restructuring tissue architecture to form physical barriers, modulating in vivo biochemical defense responses, activating molecular immune pathways, and shaping rhizosphere microbial community composition. In particular, calcium, boron, and silicon strengthen cell-wall structure and elevate defense enzyme activities, thereby substantially reducing disease incidence. Romera et al. [27] showed that microorganisms that induce systemic resistance (ISR) can trigger iron-deficiency responses in dicotyledonous plants. This finding indicates that the ISR-mediated disease-resistance pathway overlaps with the plant iron-response pathway and shares key regulators, including ethylene and the transcription factor MYB72. It also shows that mineral nutrient metabolism is tightly linked to pathogen defense and can function dually as a biofertilizer and a biopesticide. The results of this study align with the foregoing conclusions. Rootstock–scion combinations exhibiting a lower disease index accumulated significantly higher concentrations of mineral nutrients, such as potassium and boron, in leaves and showed thicker cell walls, intact cuticle structures, and more compact anatomical tissues. Together, these two mechanisms elevated overall disease resistance. No indicators in this experiment exhibited trends that contradicted previous studies. Variation in leaf traits among rootstock–scion combinations modulates Valsa canker resistance via two principal pathways: physical defense and biochemical defense.

Leaf photosynthetic performance is a key determinant of tree disease resistance. Photosynthetic efficiency directly controls carbohydrate accumulation and the synthesis rate of defense compounds in fruit trees, and it interacts synergistically with mineral nutrient metabolism and branch tissue architecture [28,29]. Rootstock–scion combinations that combine high photosynthetic capacity with balanced mineral nutrient accumulation maintain compact branch and leaf tissue structure, thus contributing to a lower disease index in the plants. In summary, leaf mineral–nutrient accumulation, leaf anatomical structure, and photosynthetic-physiological traits are important physiological and morphological indicators for evaluating Valsa canker resistance among apple rootstock–scion combinations. When considered alongside branch anatomy and rootstock–scion compatibility, these parameters provide key auxiliary indices for comprehensively assessing the disease resistance of rootstock–scion combinations.

This study assessed Valsa canker in apples by recording disease symptoms under natural field conditions. We calculated a disease index from field disease grading statistics, which sufficed for comparative analysis of resistance phenotypes among different rootstock–scion combinations and effectively captured differences in field resistance. However, we did not isolate, purify, or culture Valsa canker in vitro, nor did we perform molecular identification of the pathogen or verification experiments that test interactions between the pathogen and relevant traits; this omission represents a limitation of the work. Consequently, the physiological and anatomical traits identified here are confirmed only as significantly correlated with field Valsa canker severity and are insufficient to explain the intrinsic regulatory or core resistance mechanisms of rootstock–scion defenses. Future studies should include artificial inoculation, molecular identification of the pathogen, and functional gene validation to elucidate the deeper regulatory mechanisms of Valsa canker resistance in apple rootstock–scion combinations.

## 4. Materials and Methods

### 4.1. Plant Materials

The rootstocks used in this study were *M. robusta* Rehd., *M. baccata* Borkh., *M. hupehensis* Rehd., Qingzhen No.1, and Qingzhen No.2. The scion cultivars were ‘Yueyanghong’, ‘Harlikar’, and ‘Qiufuhong’. The rootstock–scion combinations are listed in Table 7. The experiment was carried out in the apple orchard of the Liaoning Institute of Pomology. The site has a warm temperate maritime monsoon climate, with a mean temperature of 6.7 °C in January and 23.7 °C in July. Annual precipitation is approximately 750 mm, concentrated mainly from July to September. Annual sunshine totals 2450 h, and the frost-free period lasts 170–186 days.

The experimental orchard was established in 2019 with a planting spacing of 2.5 m × 4.0 m. Trees were arranged in rows in a north–south orientation. Each rootstock–scion combination occupied three consecutive rows, forming one planting block. For sampling, marginal trees at the beginning and end of each row were excluded; 30 trees were then randomly selected and divided into three groups of 10 trees each, serving as three biological replicates. The three groups of 10 trees used as biological replicates corresponded directly to the three individual rows. All experimental trees received uniform water and fertilizer management. The plot soil was clay loam, with soil organic matter content (mass fraction, the same below) of 1.33%, total nitrogen of 0.64%, available phosphorus of 70.3 mg·kg^−1^, and available potassium of 107 mg·kg^−1^.

### 4.2. Determination Indexes and Methods

#### 4.2.1. Growth Indices

Diameters of the rootstock (RD), scion (SD), and graft union (GD), along with leaf length (LL), leaf width (LW), and leaf thickness (LT), were measured with a digital caliper (Mitutoyo, Kawasaki, Japan) 5 cm above and below the graft union. Leaf area (LA) was calculated using ImageJ 1.53a software, and the field compatibility constant (FCC) and rootstock–scion ratio (RSR) were determined following previous methods [30]. An FCC value of approximately 2.0 indicates good compatibility of the rootstock–scion combination. Values substantially greater than 2.0 reflect an excessively large rootstock diameter and uncoordinated growth at the graft site, while values substantially less than 2.0 reflect an excessively large scion diameter and similarly poor compatibility. Each combination consisted of three biological replicates, with three technical replicates per biological replicate.

(1) FCC=C/A+(C+A)/2B

(2) RSR=C/A

A = diameter at 5 cm above the graft union; B = diameter at the graft union; C = diameter at 5 cm below the graft union.

#### 4.2.2. Physiological Indices

In July 2025, measurements were made between 9:00 and 12:00. For each plant, the 5th functional leaf at the same position was selected at random. Chlorophyll content (SPAD value) was measured with a SPAD-502 chlorophyll meter (Konica minolta, Tokyo, Japan); each combination consisted of three biological replicates with ten technical replicates per treatment. A photosynthesis system (Yaxin-1105, Beijing, China) was used to record chlorophyll fluorescence and photosynthetic parameters, including initial fluorescence (F_0_), maximum fluorescence (F_m_), potential photochemical activity (F_V_/F_0_), maximum photochemical efficiency (F_V_/F_m_), performance index of light energy absorption (PI_ABS), net photosynthetic rate (Pn), stomatal conductance (Gs), transpiration rate (Tr), and intercellular CO_2_ concentration (Ci). Thirty mature leaves at the same leaf position were sampled for further assays. Leaf relative electrolyte conductivity (REC) was determined following the method of Cao Jiankang [31]. Mineral element contents were measured after microwave digestion by inductively coupled plasma mass spectrometry (ICP-MS). Three biological replicates were performed for each measurement. all determinations were conducted in triplicate.

#### 4.2.3. Disease Resistance Indicators

Investigations on plants of different rootstock–scion combinations were conducted in the orchard during the peak period of apple Valsa canker occurrence in spring. Valsa canker symptoms were assessed based on field visual observation. For each combination, 30 trees were surveyed successively by row; the disease-infected parts, the number of current canker lesions, and the area of canker lesions of each tree were recorded to determine the disease severity. The grading criteria for disease severity from Grade 0 to Grade 4 are shown in Table 8, and the disease incidence and disease index were calculated accordingly [32].

(3) $$\mathrm{Disease}\;\mathrm{index}\;=\frac{\sum \left(\mathrm{Plants}\;\mathrm{per}\;\mathrm{disease}\;\mathrm{grade}\times \mathrm{Representative}\;\mathrm{value}\;\mathrm{of}\;\mathrm{each}\;\mathrm{grade}\right)}{\mathrm{Total}\;\mathrm{plants}\times \mathrm{Highest}\;\mathrm{grade}\;\mathrm{value}}\times 100\%$$

(4) $$\mathrm{Incidence}\;\mathrm{rate}=\frac{\mathrm{Number}\;\mathrm{of}\;\mathrm{diseased}\;\mathrm{plants}}{\mathrm{Total}\;\mathrm{number}\;\mathrm{of}\;\mathrm{investigated}\;\mathrm{plants}}\times 100\%$$

#### 4.2.4. Observation of Anatomical Structures

For each rootstock–scion combination, healthy one-year-old shoots were collected and fixed in FAA. Samples were dehydrated through a graded ethanol series (Sinopharm Chemical Reagent Co., Ltd., Shanghai, China), cleared with xylene (Sinopharm Chemical Reagent Co., Ltd., Shanghai, China), and embedded by conventional paraffin methods. Sections were stained with safranin and fast green and mounted for observation. Microscopy was used to analyze anatomical features, including pith, xylem, cambium, phloem, and vessel diameter. Three biological replicates were prepared per combination, with three paraffin sections per replicate and three fields of view examined for each section.

### 4.3. Data Analysis

Statistical analyses were performed with SPSS 26.0. One-way analysis of variance (ANOVA) was followed by multiple comparisons using Duncan’s new multiple-range test (*p* < 0.05) to assess differences among scion–rootstock combinations. Two-way ANOVA was used to partition scion and rootstock effects at *p* < 0.0001. Correlation analyses were also conducted. The mean values of three biological replicates for each parameter were used for correlation analyses. All figures and graphs were produced with Origin 2019.

## 5. Conclusions

Using 15 apple rootstock–scion combinations, this study conducted a field evaluation of the effects of rootstock, scion, and their interaction on tree growth, leaf photosynthesis, mineral nutrient uptake, and resistance to Valsa canker. Rootstock effects predominated for tree-growth traits; rootstock significantly influenced rootstock diameter, scion diameter, rootstock–scion ratio, and field compatibility constant, whereas scion chiefly determined graft–union diameter. The rootstock–scion interaction mainly affected rootstock diameter and graft–union diameter, with smaller effects on rootstock–scion ratio and field compatibility constant. Leaf growth was only weakly modulated by these three factors; scion significantly affected leaf width, and scion together with the rootstock–scion interaction jointly regulated leaf area. For photosynthetic indices, scion effects were most pronounced; scion had stronger influences on intercellular CO_2_ concentration, relative electrical conductivity, SPAD value, and photosynthetic performance index (PI_ABS) than the rootstock–scion interaction, while rootstock had limited impact on these parameters. Mineral element accumulation showed distinct regulatory patterns: K and Ca were mainly controlled by scion, Mg was primarily regulated by rootstock, and N and P were not significantly affected by rootstock, scion, or their interaction.

Apple resistance to Valsa canker showed clear rootstock–scion combination specificity, with marked effects attributable to both rootstock and scion. ‘Yueyanghong’ exhibited the strongest overall disease resistance, while ‘Harlikar’/Qingzhen No.2 and ‘Qiufuhong’/*M. baccata* Borkh. displayed the most severe symptoms. Graft compatibility between rootstock and scion is essential for sustaining normal tree growth and baseline disease resistance. Combinations with a compatibility constant near 2.0 and a balanced rootstock–scion ratio had well-coordinated vigor and lower disease severity. Higher leaf net photosynthetic rates, stable cell membrane integrity, and appropriate potassium levels were associated with Valsa canker incidence, whereas excessive accumulation of trace elements such as Fe, Mn, and Zn may contribute to disease development. Phloem thickness and cell density in one-year-old branches correlated significantly with disease resistance. Qingzhen series rootstocks increased the thickness of lignified branch structures, and the pith cell density that emerged may represent a resistance-related trait. In summary, Valsa canker resistance in apple rootstock–scion combinations reflects the combined influences of compatibility, photosynthetic physiology, mineral nutrition, and branch anatomical traits. Leaf net photosynthetic rate, relative electrical conductivity, branch phloem thickness, cell density, and field compatibility constant can serve as preliminary evaluation indices for resistance. These findings provide a theoretical foundation and practical guidance for selecting disease-resistant apple rootstock–scion combinations and for developing high-quality cultivation practices.

## Figures and Tables

**Table 1:** Effects of different rootstock–scion combinations on plant growth.

Scion	Rootstock	RD/mm	SD/mm	GD/mm	RSR	FCC
Yueyanghong	1	81.57 ± 1.33 ^bc^	82.57 ± 1.75 ^b^	93.91 ± 1.17 ^a^	0.98 ± 0.02 ^a^	1.87 ± 0.07 ^b^
	2	101.92 ± 1.30 ^a^	92.66 ± 1.69 ^ab^	97.06 ± 2.08 ^a^	1.10 ± 0.03 ^a^	2.10 ± 0.03 ^a^
	3	82.16 ± 3.55 ^bc^	106.01 ± 2.24 ^a^	107.15 ± 4.26 ^a^	0.77 ± 0.03 ^b^	1.66 ± 0.04 ^c^
	4	91.01 ± 3.42 ^ab^	92.56 ± 3.08 ^ab^	104.50 ± 2.53 ^a^	0.99 ± 0.06 ^a^	1.87 ± 0.02 ^b^
	5	73.49 ± 2.88 ^c^	75.22 ± 4.20 ^b^	88.30 ± 3.04 ^a^	0.97 ± 0.04 ^a^	1.82 ± 0.02 ^b^
Harlikar	1	96.01 ± 3.86 ^a^	102.21 ± 3.84 ^ab^	114.86 ± 3.24 ^a^	0.93 ± 0.02 ^c^	1.80 ± 0.02 ^cd^
	2	78.34 ± 2.80 ^c^	64.13 ± 1.79 ^d^	82.60 ± 2.44 ^c^	1.22 ± 0.02 ^a^	2.08 ± 0.01 ^a^
	3	86.14 ± 3.89 ^bc^	105.57 ± 3.91 ^a^	105.03 ± 3.61 ^a^	0.81 ± 0.01 ^d^	1.72 ± 0.02 ^d^
	4	93.33 ± 2.73 ^ab^	93.26 ± 1.84 ^b^	94.54 ± 2.65 ^b^	1.00 ± 0.01 ^bc^	1.98 ± 0.01 ^b^
	5	86.85 ± 2.21 ^bc^	80.72 ± 2.61 ^c^	113.16 ± 5.27 ^a^	1.07 ± 0.05 ^b^	1.82 ± 0.04 ^c^
Qiufuhong	1	90.63 ± 2.55 ^a^	108.97 ± 4.18 ^a^	127.36 ± 5.35 ^a^	0.83 ± 0.01 ^bc^	1.62 ± 0.06 ^a^
	2	94.68 ± 3.93 ^a^	90.64 ± 2.41 ^bc^	129.95 ± 6.40 ^a^	1.04 ± 0.01 ^a^	1.77 ± 0.10 ^a^
	3	87.14 ± 4.32 ^a^	110.03 ± 3.47 ^a^	118.24 ± 7.20 ^a^	0.79 ± 0.04 ^c^	1.63 ± 0.01 ^a^
	4	87.04 ± 1.89 ^a^	96.39 ± 2.71 ^b^	114.04 ± 4.91 ^a^	0.90 ± 0.05 ^b^	1.70 ± 0.04 ^a^
	5	76.73 ± 0.73 ^b^	85.63 ± 5.94 ^c^	117.74 ± 8.36 ^a^	0.89 ± 0.02 ^b^	1.59 ± 0.05 ^a^
*p*	Scion	0.563	0.002 *	1.00 × 10^−6^ ***	2.30 × 10^−5^ ***	5.32 × 10^−7^ ***
	Rootstock	1.17 × 10^−5^ ***	5.00 × 10^−7^ ***	0.298	3.00 × 10^−7^ ***	1.02 × 10^−6^ ***
	Interaction	4.40 × 10^−5^ ***	0.001	0.004 *	0.091	0.087

Note: Lowercase letters indicate significant differences at the *p* < 0.05 level. One-way ANOVA was used for each Scion identifier. 1: Qingzhen No.1, 2: Qingzhen No.2, 3: *M. baccata* Borkh., 4: *M. robusta* Rehd., 5: *M. hupehensis* Rehd. *, and *** indicate the significance at *p* < 0.01 and *p* < 0.0001 with two-way ANOVA, respectively.

**Table 2:** Effects of different rootstock–scion combinations on leaf growth.

Scion	Rootstock	LT/mm	LL/cm	LW/cm	LA/cm^2^
Yueyanghong	1	0.34 ± 0.04 ^a^	9.58 ± 0.68 ^a^	5.35 ± 0.50 ^a^	36.81 ± 1.35 ^a^
	2	0.32 ± 0.01 ^a^	8.27 ± 0.21 ^b^	4.18 ± 0.32 ^c^	24.57 ± 2.45 ^c^
	3	0.33 ± 0.03 ^a^	8.39 ± 0.52 ^b^	4.41 ± 0.30 ^bc^	27.68 ± 3.43 ^bc^
	4	0.34 ± 0.03 ^a^	8.74 ± 0.43 ^ab^	5.05 ± 0.37 ^ab^	31.74 ± 3.72 ^b^
	5	0.32 ± 0.05 ^a^	8.02 ± 0.53 ^b^	4.47 ± 0.19 ^bc^	26.13 ± 1.07 ^c^
Harlikar	1	0.34 ± 0.03 ^a^	7.90 ± 0.66 ^a^	4.79 ± 0.13 ^a^	27.55 ± 0.61 ^a^
	2	0.31 ± 0.02 ^a^	7.61 ± 0.44 ^a^	4.92 ± 0.27 ^a^	28.41 ± 2.65 ^a^
	3	0.33 ± 0.04 ^a^	8.84 ± 0.89 ^a^	4.59 ± 0.11 ^a^	28.38 ± 2.06 ^a^
	4	0.36 ± 0.03 ^a^	8.15 ± 0.18 ^a^	4.76 ± 0.26 ^a^	28.64 ± 1.87 ^a^
	5	0.33 ± 0.01 ^a^	8.02 ± 1.21 ^a^	4.78 ± 0.43 ^a^	28.74 ± 4.81 ^a^
Qiufuhong	1	0.34 ± 0.03 ^a^	8.05 ± 0.72 ^ab^	5.68 ± 0.12 ^a^	35.16 ± 2.21 ^a^
	2	0.34 ± 0.02 ^a^	7.92 ± 0.05 ^ab^	5.88 ± 0.60 ^a^	35.14 ± 4.86 ^a^
	3	0.34 ± 0.04 ^a^	7.67 ± 0.52 ^b^	4.87 ± 0.58 ^a^	28.06 ± 3.59 ^a^
	4	0.35 ± 0.03 ^a^	7.72 ± 0.23 ^ab^	5.34 ± 0.58 ^a^	30.37 ± 3.38 ^a^
	5	0.33 ± 0.04 ^a^	8.64 ± 0.30 ^a^	5.79 ± 0.88 ^a^	37.98 ± 6.40 ^a^
*p*	Scion	0.753	0.020	1.60 × 10^−5^ ***	0.001 *
	Rootstock	0.385	0.365	0.056	0.036
	Interaction	0.998	0.027	0.071	0.002 *

Note: Lowercase letters indicate significant differences at the *p* < 0.05 level. One-way ANOVA was used for each Scion identifier. 1: Qingzhen No.1, 2: Qingzhen No.2, 3: *M. baccata* Borkh., 4: *M. ro-busta* Rehd., 5: *M. hupehensis* Rehd. * and *** indicate the significance at *p* < 0.01 and *p* < 0.0001 with two-way ANOVA, respectively.

**Table 3:** Effects of different rootstock–scion combinations on leaf photosynthetic parameters.

Scion	Rootstock	Pn /μmol·m^−2^·s^−1^	Tr /mmol·m^−2^·s^−1^	Gs /mol·m^−2^·s^−1^	Ci /μmol·m^−2^·s^−1^	REC /%	SPAD
Yueyanghong	1	13.06 ± 0.48 ^b^	2.89 ± 0.15 ^a^	136.30 ± 4.72 ^ab^	282.10 ± 14.25 ^b^	0.31 ± 0.02 ^b^	59.55 ± 1.07 ^ab^
	2	15.26 ± 0.72 ^a^	2.72 ± 0.10 ^a^	151.60 ± 7.59 ^a^	277.13 ± 10.51 ^b^	0.30 ± 0.01 ^b^	60.49 ± 1.01 ^ab^
	3	14.11 ± 0.30 ^ab^	2.98 ± 0.16 ^a^	148.90 ± 1.69 ^a^	292.70 ± 8.14 ^b^	0.46 ± 0.03 ^a^	61.54 ± 1.24 ^ab^
	4	12.72 ± 1.06 ^b^	2.73 ± 0.25 ^a^	117.13 ± 6.88 ^b^	256.30 ± 15.16 ^b^	0.32 ± 0.01 ^b^	58.28 ± 1.18 ^b^
	5	16.50 ± 0.43 ^a^	2.78 ± 0.08 ^a^	145.47 ± 6.08 ^a^	336.43 ± 13.28 ^a^	0.46 ± 0.01 ^a^	62.04 ± 1.13 ^a^
Harlikar	1	9.92 ± 0.46 ^b^	2.91 ± 0.36 ^ab^	129.45 ± 4.61 ^b^	259.80 ± 3.07 ^a^	0.30 ± 0.03 ^a^	58.35 ± 1.39 ^a^
	2	7.83 ± 1.12 ^b^	2.04 ± 0.25 ^b^	72.06 ± 5.89 ^c^	256.30 ± 6.01 ^a^	0.40 ± 0.02 ^a^	59.34 ± 1.01 ^a^
	3	13.69 ± 1.17 ^a^	3.13 ± 0.34 ^a^	169.09 ± 10.16 ^a^	273.90 ± 4.89 ^a^	0.46 ± 0.05 ^a^	58.08 ± 1.30 ^a^
	4	13.92 ± 1.23 ^a^	3.78 ± 0.32 ^a^	181.34 ± 10.01 ^a^	281.36 ± 4.13 ^a^	0.31 ± 0.02 ^a^	57.5 ± 1.50 ^a^
	5	13.08 ± 0.63 ^a^	2.81 ± 0.26 ^ab^	127.22 ± 9.55 ^b^	295.40 ± 11.55 ^a^	0.34 ± 0.07 ^a^	58.13 ± 1.03 ^a^
Qiufuhong	1	13.73 ± 1.06 ^a^	3.68 ± 0.15 ^a^	152.23 ± 7.34 ^a^	277.70 ± 8.10 ^a^	0.67 ± 0.02 ^a^	57.15 ± 1.25 ^bc^
	2	10.82 ± 0.63 ^b^	2.82 ± 0.11 ^a^	108.20 ± 3.96 ^b^	220.80 ± 11.57 ^b^	0.53 ± 0.03 ^bc^	55.34 ± 0.85 ^c^
	3	13.08 ± 0.74 ^ab^	2.75 ± 0.53 ^a^	131.04 ± 9.09 ^ab^	224.36 ± 6.22 ^b^	0.49 ± 0.01 ^c^	58.81 ± 1.08 ^ab^
	4	11.81 ± 1.01 ^ab^	3.18 ± 0.35 ^a^	124.50 ± 8.81 ^b^	211.73 ± 9.84 ^b^	0.63 ± 0.03 ^ab^	57.54 ± 1.04 ^abc^
	5	11.70 ± 0.76 ^ab^	2.63 ± 0.37 ^a^	113.52 ± 10.62 ^b^	215.67 ± 10.57 ^b^	0.56 ± 0.04 ^abc^	60.73 ± 1.19 ^a^
*p*	Scion	0.26	0.379	0.177	7 × 10^−6^ ***	7.21 × 10^−7^ ***	1.79 × 10^−17^ ***
	Rootstock	0.382	0.041	0.366	0.186	0.289	0.02
	Interaction	0.044	0.38	0.74	0.058	2.90 × 10^−4^ ***	3.15 × 10^−12^ ***

Note: Lowercase letters indicate significant differences at the *p* < 0.05 level. One-way ANOVA was used for each Scion identifier. 1: Qingzhen No.1, 2: Qingzhen No.2, 3: *M. baccata* Borkh., 4: *M. ro-busta* Rehd., 5: *M. hupehensis* Rehd. *** indicate the significance at *p* < 0.0001 with two-way ANOVA, respectively.

**Table 4:** Leaf chlorophyll fluorescence parameters under different rootstock–scion combinations.

Scion	Rootstock	F_0_	F_m_	F_V_	F_V_/F_m_	PI_ABS
Yueyanghong	1	227.33 ± 9.21 ^a^	1292.33 ± 75.36 ^a^	1064.86 ± 70.39 ^a^	0.82 ± 0.01 ^a^	4.39 ± 0.08 ^a^
	2	216.00 ± 7.21 ^a^	1065.33 ± 90.80 ^ab^	862.47 ± 91.79 ^ab^	0.80 ± 0.02 ^a^	2.05 ± 0.38 ^b^
	3	215.67 ± 7.17 ^a^	1014.33 ± 67.82 ^b^	800.61 ± 73.73 ^b^	0.78 ± 0.02 ^a^	2.54 ± 0.29 ^b^
	4	210.07 ± 4.93 ^a^	1101.67 ± 65.33 ^ab^	889.83 ± 62.13 ^ab^	0.80 ± 0.02 ^a^	3.84 ± 0.26 ^a^
	5	211.67 ± 5.33 ^a^	1052.33 ± 66.12 ^ab^	840.26 ± 67.76 ^ab^	0.79 ± 0.01 ^a^	2.62 ± 0.32 ^b^
Harlikar	1	270.33 ± 2.67 ^a^	1401.23 ± 73.70 ^a^	1166.74 ± 42.94 ^a^	0.83 ± 0.01 ^a^	5.84 ± 0.53 ^a^
	2	218.33 ± 11.07 ^bc^	1163.79 ± 59.47 ^a^	945.88 ± 51.03 ^a^	0.81 ± 0.01 ^a^	4.75 ± 0.72 ^a^
	3	212.67 ± 6.17 ^c^	1236.00 ± 39.67 ^a^	1022.28 ± 35.29 ^a^	0.82 ± 0.01 ^a^	6.35 ± 0.34 ^a^
	4	218.66 ± 11.25 ^bc^	1236.60 ± 44.97 ^a^	1018.86 ± 48.16 ^a^	0.82 ± 0.01 ^a^	5.70 ± 0.29 ^a^
	5	260.33 ± 18.41 ^ab^	1288.00 ± 26.89 ^a^	1029.95 ± 31.89 ^a^	0.80 ± 0.01 ^a^	4.90 ± 0.31 ^a^
Qiufuhong	1	249.33 ± 9.52 ^a^	1344.33 ± 97.06 ^a^	1097.23 ± 74.47 ^a^	0.81 ± 0.01 ^a^	4.26 ± 0.57 ^a^
	2	203.33 ± 7.79 ^b^	1189.00 ± 54.93 ^a^	987.03 ± 47.66 ^a^	0.83 ± 0.02 ^a^	4.83 ± 0.81 ^a^
	3	196.33 ± 8.33 ^b^	1259.67 ± 44.92 ^a^	1062.76 ± 37.34 ^a^	0.84 ± 0.01 ^a^	4.92 ± 0.57 ^a^
	4	216.33 ± 8.41 ^ab^	1347.00 ± 69.86 ^a^	1131.89 ± 63.46 ^a^	0.84 ± 0.02 ^a^	4.38 ± 0.27 ^a^
	5	204.67 ± 8.95 ^b^	1215.67 ± 82.14 ^a^	1002.57 ± 83.14 ^a^	0.82 ± 0.01 ^a^	4.52 ± 0.63 ^a^
*p*	Scion	0.284	0.018	0.014	0.028	9.31 × 10^−5^ ***
	Rootstock	0.245	0.750	0.764	0.495	0.514
	Interaction	0.151	0.200	0.196	0.266	0.155

Note: Lowercase letters indicate significant differences at the *p* < 0.05 level. One-way ANOVA was used for each Scion identifier. 1: Qingzhen No.1, 2: Qingzhen No.2, 3: *M. baccata* Borkh., 4: *M. ro-busta* Rehd., 5: *M. hupehensis* Rehd. *** indicate the significance at *p* < 0.0001 with two-way ANOVA, respectively.

**Table 5:** Effects of different rootstock–scion combinations on macronutrient contents in leaves.

Scion	Rootstock	N/mg·g^−1^	P/mg·g^−1^	K/mg·g^−1^	Ca/mg·g^−1^	Mg/mg·g^−1^
Yueyanghong	1	25.63 ± 2.79 ^a^	2.12 ± 0.63 ^a^	13.26 ± 1.73 ^a^	13.45 ± 1.18 ^a^	4.43 ± 0.43 ^a^
	2	27.99 ± 1.26 ^a^	1.79 ± 0.55 ^ab^	13.71 ± 0.96 ^a^	12.51 ± 1.73 ^ab^	4.39 ± 0.57 ^a^
	3	24.73 ± 0.84 ^a^	1.82 ± 0.24 ^ab^	12.81 ± 0.83 ^a^	11.96 ± 0.82 ^b^	3.34 ± 0.46 ^a^
	4	26.41 ± 2.64 ^a^	1.85 ± 0.51 ^ab^	12.89 ± 1.03 ^a^	10.25 ± 0.55 ^b^	3.62 ± 0.82 ^a^
	5	26.88 ± 3.11 ^a^	1.57 ± 0.37 ^b^	13.59 ± 0.57 ^a^	11.83 ± 0.71 ^b^	4.43 ± 0.73 ^a^
Harlikar	1	25.81 ± 3.42 ^a^	1.77 ± 0.31 ^a^	11.83 ± 0.61 ^b^	11.30 ± 1.43 ^a^	3.64 ± 0.55 ^a^
	2	26.01 ± 2.92 ^a^	1.85 ± 0.47 ^a^	10.98 ± 1.36 ^b^	8.93 ± 1.05 ^b^	3.03 ± 0.37 ^a^
	3	23.27 ± 1.17 ^a^	1.36 ± 0.22 ^a^	13.18 ± 0.89 ^a^	10.98 ± 1.72 ^a^	4.10 ± 0.77 ^a^
	4	23.53 ± 0.63 ^a^	1.59 ± 0.49 ^a^	10.83 ± 1.63 ^b^	8.96 ± 1.41 ^b^	3.22 ± 0.69 ^a^
	5	24.84 ± 1.54 ^a^	1.60 ± 0.25 ^a^	11.07 ± 1.04 ^b^	10.52 ± 0.94 ^a^	3.22 ± 0.47 ^a^
Qiufuhong	1	25.13 ± 1.71 ^a^	1.78 ± 0.83 ^a^	12.14 ± 0.73 ^ab^	14.44 ± 1.73 ^a^	4.48 ± 0.88 ^a^
	2	26.64 ± 0.94 ^a^	1.68 ± 0.72 ^a^	11.65 ± 1.42 ^b^	13.65 ± 1.45 ^a^	4.01 ± 0.32 ^a^
	3	27.02 ± 2.29 ^a^	1.66 ± 0.14 ^a^	13.26 ± 1.29 ^a^	12.78 ± 0.83 ^ab^	3.87 ± 0.95 ^a^
	4	26.26 ± 1.51 ^a^	1.46 ± 0.33 ^a^	13.05 ± 0.77 ^a^	11.24 ± 1.39 ^b^	3.67 ± 0.45 ^a^
	5	27.62 ± 1.88 ^a^	1.52 ± 0.51 ^a^	13.38 ± 0.84 ^a^	12.40 ± 1.44 ^ab^	3.85 ± 0.74 ^a^
*p*	Scion	0.011	0.418	1.32 × 10^−4^ ***	1.51 × 10^−5^ ***	0.010
	Rootstock	0.036	0.071	0.007 *	1.12 × 10^−3^ ***	2.06 × 10^−4^ ***
	Interaction	0.011	0.146	0.007 *	0.018	0.012

Note: Lowercase letters indicate significant differences at the *p* < 0.05 level. One-way ANOVA was used for each Scion identifier. 1: Qingzhen No.1, 2: Qingzhen No.2, 3: *M. baccata* Borkh., 4: *M. ro-busta* Rehd., 5: *M. hupehensis* Rehd. * and *** indicate the significance at *p* < 0.01 and *p* < 0.0001 with two-way ANOVA, respectively.

**Table 6:** Effects of different rootstock–scion combinations on micronutrient contents in leaves.

Scion	Rootstock	Fe/mg·kg^−1^	Zn/mg·kg^−1^	Mn/mg·kg^−1^	Cu/mg·kg^−1^	B/mg·kg^−1^
Yueyanghong	1	141.21 ± 8.43 ^b^	13.64 ± 2.44 ^b^	91.65 ± 4.93 ^c^	10.74 ± 1.72 ^b^	18.64 ± 2.30 ^b^
	2	177.99 ± 11.40 ^a^	19.72 ± 3.82 ^a^	172.80 ± 5.95 ^ab^	19.26 ± 2.38 ^a^	27.71 ± 1.94 ^a^
	3	138.30 ± 9.04 ^b^	19.54 ± 2.91 ^a^	117.62 ± 7.32 ^b^	9.39 ± 1.07 ^b^	29.81 ± 3.85 ^a^
	4	124.43 ± 6.48 ^b^	21.10 ± 2.39 ^a^	158.10 ± 7.99 ^ab^	8.72 ± 0.83 ^b^	23.19 ± 3.29 ^ab^
	5	121.33 ± 5.25 ^b^	17.44 ± 2.71 ^ab^	194.98 ± 12.53 ^a^	6.75 ± 0.52 ^c^	20.47 ± 2.51 ^ab^
Harlikar	1	152.46 ± 7.93 ^a^	23.97 ± 2.83 ^b^	266.76 ± 10.08 ^a^	12.91 ± 1.94 ^a^	30.30 ± 3.33 ^ab^
	2	146.55 ± 7.44 ^a^	34.83 ± 3.96 ^a^	209.36 ± 8.51 ^b^	10.42 ± 1.33 ^b^	27.49 ± 2.64 ^b^
	3	128.83 ± 6.71 ^b^	21.71 ± 3.77 ^b^	179.41 ± 14.40 ^b^	9.85 ± 1.78 ^b^	34.67 ± 3.92 ^a^
	4	139.47 ± 9.52 ^b^	19.17 ± 3.01 ^b^	190.85 ± 7.43 ^b^	7.63 ± 0.88 ^b^	25.11 ± 2.47 ^b^
	5	147.47 ± 3.90 ^a^	14.31 ± 2.11 ^c^	221.68 ± 11.42 ^ab^	6.55 ± 0.91 ^b^	25.88 ± 1.74 ^b^
Qiufuhong	1	118.32 ± 5.83 ^b^	24.69 ± 3.25 ^a^	79.44 ± 5.81 ^c^	5.47 ± 0.73 ^b^	26.49 ± 2.67 ^b^
	2	215.36 ± 6.22 ^ab^	21.88 ± 2.71 ^a^	216.75 ± 10.04 ^a^	6.16 ± 0.47 ^b^	27.95 ± 3.71 ^b^
	3	224.46 ± 5.45 ^a^	23.89 ± 2.28 ^a^	152.36 ± 6.83 ^b^	12.82 ± 1.64 ^a^	38.44 ± 4.02 ^a^
	4	210.68 ± 13.74 ^ab^	23.24 ± 3.64 ^a^	126.77 ± 7.07 ^b^	5.42 ± 0.83 ^b^	31.90 ± 2.95 ^ab^
	5	226.52 ± 9.51 ^a^	24.46 ± 2.75 ^a^	233.45 ± 8.43 ^a^	15.12 ± 1.84 ^a^	30.92 ± 3.88 ^ab^
*p*	Scion	9.39 × 10^−6^ ***	5.02 × 10^−5^ ***	3.23 × 10^−4^ ***	1.18 × 10^−5^ ***	5.51 × 10^−4^ ***
	Rootstock	7.30 × 10^−5^ ***	8.20 × 10^−6^ ***	5.41 × 10^−4^ ***	1.00 × 10^−5^ ***	7.30 × 10^−3^ ***
	Interaction	5.23 × 10^−5^ ***	4.97 × 10^−4^ ***	7.90 × 10^−4^ ***	3.2 × 10^−6^ ***	3.90 × 10^−3^ ***

Note: Lowercase letters indicate significant differences at the *p* < 0.05 level. One-way ANOVA was used for each Scion identifier. 1: Qingzhen No.1, 2: Qingzhen No.2, 3: *M. baccata* Borkh., 4: *M. ro-busta* Rehd., 5: *M. hupehensis* Rehd. *** indicate the significance at *p* < 0.0001 with two-way ANOVA, respectively.

**Table 7:** List of rootstock–scion combinations.

Scion	Rootstock	The Utilization Methods of Rootstocks	Tree Age
Yueyanghong	Qingzhen No.1	Dwarfing rootstock	7
	Qingzhen No.2		7
	*M. baccata* Borkh.	Standard rootstock	7
	*M. robusta* Rehd.		7
	*M. hupehensis* Rehd.		7
Harlikar	Qingzhen No.1	Dwarfing rootstock	7
	Qingzhen No.2		7
	*M. baccata* Borkh.	Standard rootstock	7
	*M. robusta* Rehd.		7
	*M. hupehensis* Rehd.		7
Qiufuhong	Qingzhen No.1	Dwarfing rootstock	7
	Qingzhen No.2		7
	*M. baccata* Borkh.	Standard rootstock	7
	*M. robusta* Rehd.		7
	*M. hupehensis* Rehd.		7

**Table 8:** Classification of apple Valsa canker severity.

Grade	Valsa canker Symptoms
Grade 0	No disease symptoms on branches and trunks
Grade 1	Several small lesions or 1–2 large lesions (approximately 15 cm in length) were observed on trees. Branches and trunks remained intact, with no obvious influence on tree vigor.
Grade 2	Multiple diseased scars or 3–4 large lesions on thick branches or trunks were observed on trees. The trunk remained basically intact, but tree vigor was slightly affected.
Grade 3	Trees exhibited numerous canker scars or several large lesions (over 20 cm in length) on thick branches or trunks. One or two main branches or the central leader had been removed, resulting in significant negative effects on tree vigor and yield.
Grade 4	Trees were covered with extensive canker scars, or large/severe canker lesions occurred on trunks and thick branches. Branches and trunks were incomplete, tree vigor was extremely weak, and some trees even died.

## Data Availability

The data are contained within the article.
